# Specific mutations in the D1–D2 linker region of VCP/p97 enhance ATPase activity and confer resistance to VCP inhibitors

**DOI:** 10.1038/cddiscovery.2017.65

**Published:** 2017-11-06

**Authors:** Prabhakar Bastola, Feng Wang, Matthew A Schaich, Taiping Gan, Bret D Freudenthal, Tsui-Fen Chou, Jeremy Chien

**Affiliations:** 1 Department of Pharmacology, Toxicology and Therapeutics, University of Kansas Medical Center, Kansas City, MO 66160, USA; 2 Division of Medical Genetics, Department of Pediatrics, Harbor-UCLA Medical Center and Los Angeles Biomedical Research Institute, Torrance, CA 90502, USA; 3 Department of Biochemistry and Molecular Biology, University of Kansas Medical Center, Kansas City, MO 66160, USA; 4 Department of Cancer Biology, University of Kansas Medical Center, Kansas City, MO 66160, USA; 5 Division of Molecular Medicine, Department of Internal Medicine, University of New Mexico, Albuquerque, NM 87131, USA

## Abstract

Valosin-containing protein (VCP), together with several partner proteins, extracts ubiquitinated client proteins from E3 ligase complex and facilitates their degradation through ubiquitin–proteasome system. Therefore, it plays an important role in regulating protein quality control and various cellular pathways. Recent studies also identified VCP as a lineage-specific essential gene in ovarian cancer. An orally bioavailable VCP inhibitor, CB-5083, is currently in Phase I clinical trials because it shows therapeutic effects in multiple tumor xenograft models. However, the mechanism of resistance to CB-5083 is unknown. Here, we characterized molecular mechanism of resistance to CB-5083. Using incremental exposure to CB-5083, we established CB-5083-resistant ovarian cancer cells that showed five- to six-fold resistance *in vitro* compared with parental cells. Genomic and complementary DNA sequencing of the *VCP* coding region revealed a pattern of co-selected mutations: (1) missense mutations at codon 470 in one copy resulting in increased ATPase activity and (2) nonsense or frameshift mutations at codon 606 or codon 616 in another copy causing the loss of allele-specific expression. Unbiased molecular docking studies showed codon 470 as a putative binding site for CB-5083. Furthermore, the analysis of somatic mutations in cancer genomes from the Cancer Genome Atlas (TCGA) indicated that codon 616 contains hotspot mutations in *VCP*. Thus, identification of these mutations associated with *in vitro* resistance to VCP inhibitors may be useful as potential theranostic markers while screening for patients to enroll in clinical trials. VCP has emerged as a viable therapeutic target for several cancer types, and therefore targeting such hyperactive VCP mutants should aid in improving the therapeutic outcome in cancer patients.

## Introduction

Valosin-containing protein (also known as p97 or Cdc48 in yeast) belongs to AAA (ATPase Associated with diverse cellular Activities) family of ATPase. The protein has an N terminal domain, two ATPase domains (D1 and D2), followed by a short C terminal extension.^1–4^ These domains are linked via two linker regions between N and D1 as well as D1 and D2.^2^ While the N-terminal domain mostly interacts with substrates and co-factors, both D1 and D2 domains bind and hydrolyze ATP.^1,5^ Mutations in the D2 domain that prevent binding of ATP or abolish ATP hydrolysis result in a dominant-negative phenotype, suggesting that D2 domain is responsible for driving most functions associated with VCP, while the ATPase activity in D1 domain provides a supportive function.^6^ Recently solved high-resolution cryoelectron microscopy structure of VCP depicted a two-step ATP-driven conformational changes associated with VCP activity.^2^ The structure revealed that binding of ATP to the D2 domain brings about a conformational change to D2 and subsequently to D1–D2 linker region. This allows for ATP binding at D1 domain resulting in the movement of N-terminal region. The structure uncovers the significance of the D1–D2 linker region and demonstrates that D1–D2 inhibitors causes a steric clash, which abolished the function of VCP.^2^

VCP has been linked with various cellular processes including ubiquitin-mediated degradation,^7,8^ endoplasmic reticulum (ER)-associated degradation,^9,10^ chromatin associated degradation,^11^ protein aggregate processing,^12^ endosomal trafficking,^13^ mitochondria-associated degradation^14^ and autophagy.^15^ Although VCP has been implicated in a multitude of cellular processes, its function in the ATP-mediated extraction of unfolded proteins from the ER for degradation via proteasome has been extensively studied.^16,17^ Similarly, the role of VCP-mediated ATP hydrolysis in the unfolding of ubiquinated client proteins that are targeted for degradation by the proteasome is also well established.^18^ These functions make VCP an essential component in protein quality control (PQC). PQC has emerged as an essential factor in tumor development, and components of PQC have been proven to be valid targets for cancer therapeutics. Recent genome-wide shRNA screens identified various components of PQC, such as proteasomal subunits and VCP, as essential genes in cancer cells.^19,20^ Therefore, VCP inhibitors may serve as novel cancer therapeutics that exploit unique vulnerabilities or dependencies of cancer cells.

Several studies reported the development of VCP inhibitors and showed that these compounds induce ER stress and apoptosis in cancer cells.^21–23^ High-throughput screening of compound libraries from Maybridge Hitfinder Collection and NIH Molecular Libraries Small Molecule Repository yielded in the identification of DBeQ (*N*^2^,*N*^4^-dibenzylquinazoline-2,4-diamine) as a selective, reversible, ATP-competitive inhibitor of VCP.^21^ Subsequently, a structure–activity relationship study was performed using DBeQ and *N*-benzyl-2-(2-flurorophenyl) quinazoline-4-amine 1 as a starting point. The study resulted in the identification of ML240 with improved potency towards inhibition of D2 ATPase activity.^24^ A derivative of ML240 was later developed into CB-5083, which displayed better specificity towards VCP and more importantly showed efficacy in several tumor xenograft models when administered orally.^23,25^ Such promising results allowed for the start of two Phase I clinical trials.

Prompted by the potential application of VCP inhibitors in cancer, several studies have focused on understanding the mechanism of resistance towards VCP inhibitors. Cells resistant to CB-5083 have been shown to harbor mutations in VCP, yet details regarding the mechanism of resistance are unknown.^23^ Similarly, prolonged treatment with an allosteric VCP inhibitor, NMS-873, resulted in a heterozygous A530T mutation in *VCP*. However, these resistant cell lines did not show any cross-resistance towards CB-5083 or other VCP inhibitors.^26^ These results suggest that target alterations may represent a molecular mechanism of resistance to VCP inhibitors.

Given that CB-5083 has entered the realm of clinical trials, we set out to understand the mechanism of resistance towards this compound. Here, we established and characterized the *in vitro* mechanism of resistance to CB-5083. Our study indicates target alterations as a potential mechanism of resistance to CB-5083 *in vitro*, thereby providing a critical potentially theranostic marker that should be considered in clinical trial settings. Finally, our studies highlight the relevance for the further development of novel VCP inhibitors that can overcome resistance to existing VCP inhibitors.

## Results

### Development of CB-5083 resistant cell lines

**Figure 1:**
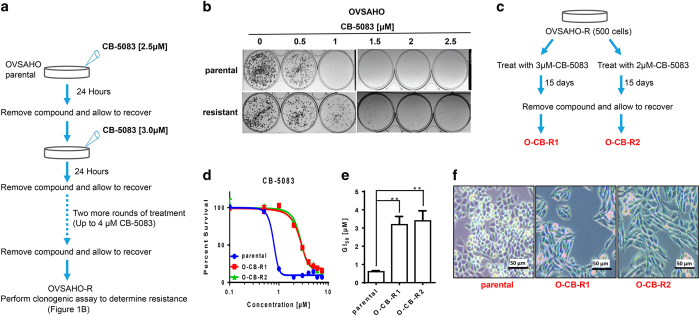
Development of CB-5083-resistant cells. (**a**) Experimental setup used to generate the initial OVSAHO-resistant cells. (**b**) Colony formation assay was performed on parental OVSAHO cells and OVSAHO-R (resistant) cells. Cells were treated with different concentrations of CB-5083 between 0.5–2.5 *μ*M for 48 h and then allowed to recover under normal media for eight additional days. (**c**) Experimental setup used to generate O-CB-R1 and O-CB-R2 from OVSAHO-R. (**d**) OVSAHO parental, O-CB-R1 and O-CB-R2 were incubated with incremental doses of CB-5083 (0.1 –10 *μ*M) for 72 h and cell viability was determined using SRB assay. Dose–response curves were generated via GraphPad Prism using four parameters nonlinear regression and the curves were constrained on top (100%) and bottom (>0%). Every point in the dose response curve represents Mean±S.E.M. taken from at least duplicate samples for all cell lines. (**e**) The bar graph represents Mean GI_50_+S.E.M. taken from three independent experiments in all cell lines. *P*-values were calculated using Student’s *t*-test. (**f**) Images of OVSAHO parental, O-CB-R1 and O-CB-R2 were taken using a Zeiss light microscope at ×100 magnification. **P value⩽0.01.

With oral VCP inhibitor CB-5083 in two Phase 1 clinical trials, we set out to understand the potential mechanism of resistance towards CB-5083. Since one of Phase 1 clinical trials for CB-5083 focuses on advanced solid tumors, we selected ovarian cancer cell line OVSAHO for our studies because it represents a solid tumor subtype with an aggressive phenotype. To understand the mechanism of resistance towards CB-5083, we established CB-5083-resistant cell lines as outlined in Figure 1a. We incubated OVSAHO cells with 2.5 *μ*M of CB-5083 for 24 h, which resulted in significant cell death (data not shown). Cells were allowed to recover in the drug-free medium for 5–10 days. Three more rounds of treatment and recovery were performed with the incremental increase of 0.5 *μ*M of CB-5083 per round (Figure 1a).

**Figure 2:**
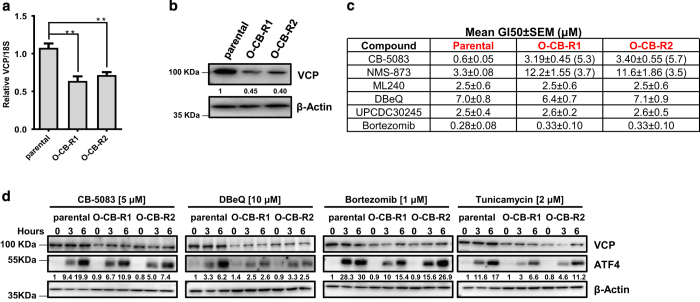
Effects on VCP/p97 mRNA and protein expression, cross-resistance and changes in unfolded protein response (UPR) in resistant cell line. (**a**) Quantitative RT-PCR was performed to evaluate the mRNA expression of VCP in OVSAHO parental, O-CB-R1 and O-CB-R2. Bar graph represents the mean relative mRNA expression+S.E.M. normalized to 18 S from three independent experiments. *P*-values were calculated using Student’s *t*-test. (**b**) Whole-cell lysates were immunoblotted and probed with the antibody against VCP to evaluate the protein expression. *β*-Actin was used as the loading control. Numbers indicate relative VCP expression normalized to the *β*-actin loading control. (**c**) Table displays Mean GI_50_±S.E.M. (*μ*M) with several VCP inhibitors and proteasome inhibitor (Bortezomib) from three independent experiments in parental and resistant cells. Numbers in the bracket for CB-5083 and NMS-873 treatment represent the drug resistance index (DRI) in resistant cells compared with parental cells. (**d**) OVSAHO parental, O-CB-R1 and O-CB-R2 were treated with CB-5083, DBeQ, bortezomib or tunicamycin at indicated concentrations. Cells were collected at 3 and 6 h time-points and whole-cell lysates were subjected to immunoblotting and probed with indicated antibodies. Numbers indicate relative ATF4 protein expression normalized to *β*-actin (loading control). **P value⩽0.01.

After four rounds of incremental exposure to CB-5083, we observed a small increase in resistance to CB-5083 in recovered cells (OVSAHO-R) compared with parental cells (Figure 1b). Next, we treated 500 OVSAHO-R cells with either 2 *μ*M CB-5083 or 3 *μ*M CB-5083 continuously for 15 days. Surviving cells were allowed to form colonies and expanded separately to get two separate populations of resistant cells (O-CB-R1 and O-CB-R2). Both surviving resistant cell populations displayed an elongated cell structure in contrast to rounded cell morphology of parental OVSAHO cells (Figure 1f and Supplementary Figure S1C). Compared with parental line, O-CB-R1 and O-CB-R2 cells lines showed 5.3- and 5.7-fold increase in resistance towards CB-5083, respectively (Figures 1d, e and 2c and Supplementary Figures S1A and B).

### Molecular characterization of resistance cell lines

We have previously shown that VCP knockdown cells were more sensitive to VCP inhibitors than VCP-proficient cells.^27^ In contrast, we observed a decrease in both mRNA as well as protein level in both resistant cell lines compared with parental (Figures 2a and b). Furthermore, we examined the cytotoxicity of several VCP inhibitors and the proteasome inhibitor bortezomib. Compared with parental cells, only NMS-873 displayed cross-resistance in O-CB-R1 and O-CB-R2 (Figure 2c and Supplementary Figure S2). Surprisingly, we were unable to observe cross-resistance towards DBeQ and ML240, which share close chemical structure to CB-5083. We also did not observe cross-resistance to the recently described allosteric VCP inhibitor UPCDC30425 as well as the proteasome inhibitor bortezomib (Figure 2c and Supplementary Figure S2).

CB-5083 treatment has been shown to induce unresolved unfolded protein response (UPR), resulting in apoptosis.^23,27^ Therefore, we evaluated UPR signaling with VCP inhibitors (CB-5083 and DBeQ), a proteasome inhibitor (bortezomib) and an ER-stress inducer (tunicamycin). Based on our cytotoxicity data, we expected CB-5083 treatment to have a reduced activation of UPR signaling in resistance cells. As expected, we observed reduced activation of ATF4 at both 3 and 6 h time-point in resistant cells (Figure 2d). We also observed the reduction of ATF4 with DBeQ, bortezomib and tunicamycin treatments (Figure 2d). Since tunicamycin and bortezomib do not bind to VCP, reduced ATF4 expression in resistant cell lines suggests that these cells might have acquired increased adaptive capacity to handle ER stress.

### CB-5083-resistant cells harbor mutations in the D1–D2 linker region of *VCP*

**Figure 3:**
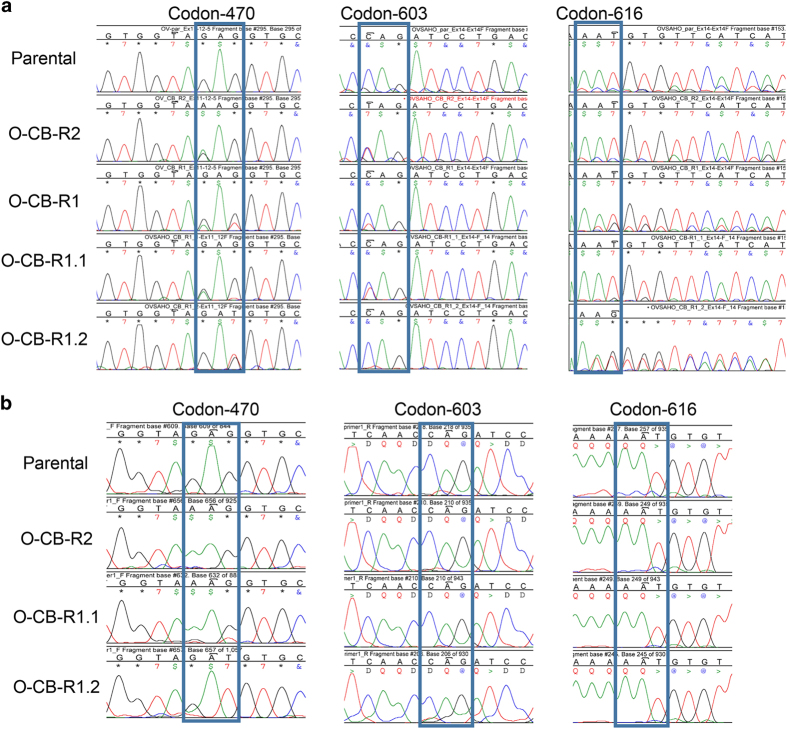
CB-5083-resistant cell lines harbor heterozygous mutations at exon 12 and exon 14. (**a**) Chromatograms displaying the region of *VCP* genomic DNA coding sequence at exon 12 and exon 14 in OVSAHO parental, O-CB-R2 and O-CB-R1 as well as two pure sub-clones O-CB-R1.1 and O-CB-R1.2 generated from O-CB-R1. Blue box marks codons 470, 603 and 616 that harbors mutations in the resistant clones. (**b**) Chromatograms displaying the region of *VCP* c-DNA in OVSAHO parental, OVSAHO-CB-R2, OVSAHO-CB-R1.1 and OVSAHO-CB-R1.2. Blue box marks codon 470, 603 and 616.

Based on previous studies indicating that CB-5083-resistant cells harbor mutations in *VCP*,^23,25^ we decided to sequence specific regions of *VCP* gene. CB-5083 has been shown to be a D2 specific inhibitor;^23,25^ therefore, we sequenced all the exons spanning the D2 domain (exons 13–16). Similarly, homozygous point mutations at the D1–D2 linker region were observed in CB-5083-resistant cells.^23^ Hence, we decided to sequence exons 11 and 12 as well. In O-CB-R2, we identified one heterozygous mutation at codon 470 (E470K, exon 12) and one heterozygous mutation at codon 603 (Q603*, exon 14) (Figure 3a). In another resistant cell line, O-CB-R1, we found two heterozygous mutations at codon 470 (E470K and E470D, exon 12), one heterozygous mutation at codon 603 (Q603*, exon 14) and one heterozygous frameshift deletion (N616Mfs, exon 14) (Figure 3a). Since both O-CB-R1 and O-CB-R2 contained a mixed population of cells and could, therefore, harbor heterogeneous mutations in VCP, we isolated several single clones from both the resistant cell populations. We generated three individual clones from O-CB-R2, and all three clones harbored a heterozygous E470K mutation in VCP (data not shown).

We also generated two individual clones from O-CB-R1 (O-CB-R1.1 and O-CB-R1.2), and observed two separate heterozygous mutations, E470K and E470D, in O-CB-R1.1 and O-CB-R1.2, respectively (Figure 3a). O-CB-R1.1 cells also contained a heterozygous nonsense mutation at codon 603 (Q603*, exon 14). In contrast, O-CB-R1.2 cells contained a heterozygous frameshift deletion at codon 616 (N616Mfs*63, exon 14) (Figure 3a). Similar to O-CB-R1, both O-CB-R1.1 and O-CB-R1.2 displayed a significant increase in GI_50_ compared with parental OVSAHO when treated with CB-5083 (Supplementary Figure S4A). In addition, analysis of somatic mutations in *VCP* in tumor samples from TCGA data set indicated that frameshift mutations at codon 616 in *VCP* are frequently observed in cancer samples (Supplementary Figures S4B and C). Previous study identified several homozygous point mutations along the D1–D2 linker region (codons 472, 473 and 474) in CB-5083-resistant cells.^23^ The clusters of mutations within this region further highlights the importance of D1–D2 linker in the mechanism of resistance to VCP inhibitors.

To determine if heterozygous mutations at codons 470 and 603/616 are compound mutations present in both alleles or mutations present separately in two alleles, we decided to sequence the cDNA. In contrast to DNA sequencing results, we detected homozygous mutations at codon 470 while no mutation was detected at codon 603 or 616 in the cDNA (Figure 3b). These results suggest that mutations at codons 470 and 603/616 are not compound mutations present in the same allele. In fact, these results suggest the first set of mutations at codon 470 (E470K or E470D) are present in one *VCP* allele while another set of mutations at codons 603 (Q603*) or 616 (N616Mfs*63) are present in the other *VCP* allele. Although VCP mRNA with E470K or E470D mutations is expressed and can be detected in cDNA sequencing as homozygous mutations, the VCP mRNA with either Q603* or N616Mfs* 63 mutations is not expressed because of nonsense-mediated decay, and therefore they are not detected in the cDNA sequencing.

### E470K and E470D mutants show higher ATPase activity and increased resistance than wild-type VCP

**Figure 4:**
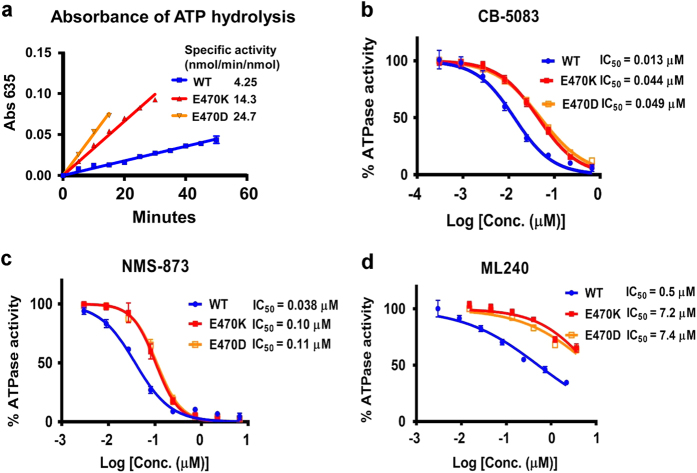
Mutations at codon 470 enhance VCP activity and attenuate VCP inhibitors function. (**a**) Time-dependent experiment with wild-type (WT) VCP/p97 and two E470 mutants to determine their specific ATPase activities. Specific ATPase activity of VCP proteins presented in the figure was measured in nmol/min/nmol. (**b**–**d**) Titration curves of CB-5083, NMS-873 and ML240 for WT, E470K and E470D VCP proteins. IC_50_ (*μ*M) was calculated using GraphPad Prism with the equation [log(agonist) *versus* response−Variable slope (four parameters)].

To determine the effect of specific missense mutations on VCP ATPase activity, we performed site-directed mutagenesis of a bacterial expression construct containing VCP as previously described.^28^ The list of plasmids used to purify VCP/p97 proteins is shown in Supplementary Figure S5C. Wild type and two mutant form of VCP proteins were purified according to our published procedure,^28^ and Biomol Green reagent (a modified Malachite Green assay) was used to determine ATPase activity. Figure 4a demonstrated that E470D and E470K have elevated ATPase activity, about 5.8- and 3.4-fold, respectively. Next, we determined *in vitro* IC_50_ of CB-5083, NMS-873, ML240, ML241 and DBeQ (Figures 4b–d and Supplementary Figures S5A and B). Our result indicated that CB-5083 was about 3.4- to 3.8-fold less active in inhibiting E470 mutants than WT VCP and interestingly, ML240 was about 15-fold less active (Figures 4b and d). The allosteric inhibitor NMS-873 was about 2.6–2.9-fold less active toward E470 mutant (Figure 4c). Increased IC_50_ in E470 mutants was also observed with ML241 and DBeQ (Supplementary Figures S5A and B). These results show E470 mutants have increased ATPase activity and resistance to a broad spectrum of VCP inhibitors.

### Mutation at E470 may affect binding to CB-5083 and NMS-873

**Figure 5:**
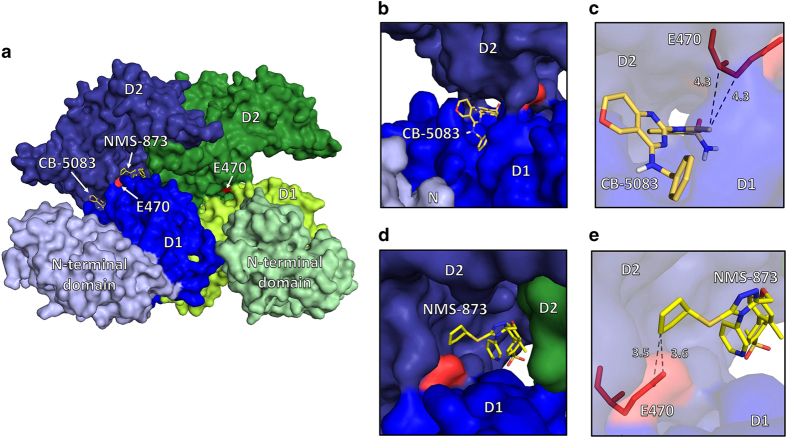
Molecular docking of CB-5083 and NMS-873 into VCP. (**a**) A structural overview of two subunits of the VCP hexamer, with one in shades of blue and one in shades of green. The lightest shade represents the N terminal domain, the medium shade represents the D1 ATPase domain and the darkest shade represents the D2 ATPase domain, as labeled. The mutation site E470 is labeled and in red. The binding sites near the D1–D2 linker of CB-5083 and NMS-873 are labeled. (**b**) The primary binding mode of CB-5083, in the interface between the D1 and D2 subdomains. (**c**) A close-up view of the binding site of CB-5083, with distances to E470 marked. (**d**) The primary binding mode of NMS-873, at the interface of two VCP subunits and the D1–D2 linker, which is also in close proximity to the mutation site E470. (**e**) A close-up view of the primary binding mode of NMS-873, with distances to the mutation site marked in Angstrom.

To determine the binding site of CB-5083, and therefore possible impacts of E470 mutations, we utilized the molecular docking program AutoDock Vina with a 2.4 Å hexameric structure of VCP (PDB code 5FTK) (Figure 5a).^2,29^ A search for binding sites of CB-5083 was performed on a hexameric VCP with a 30×30×30 Å^3^ cubic region oriented near the mutation site while keeping the D2 active site of the enzyme within the search box. Two thermodynamically similar binding modes were observed: one was at the D1–D2 interface with the methyl group from the benzimidazole ring in contact with E470. The carbon molecule of the attached methyl group is 4.3 Å from both the carbon two and three of E470 (Figures 5b and c). The calculated free energy for ligand binding of that mode was −8.9 kcal/mol. A previous docking study observed binding of CB-5083 at the ATP binding site of D2.^25^ However, the search parameters used were centered on the D2 active site of VCP and did not include the D1–D2 interface region.^25^

Similar to the previous study, we also observed an additional binding pocket in the active site of the D2 domain with similar thermodynamic favorability of binding (−9.5 kcal/mol), suggesting CB-5083 can bind at both the D1–D2 linker and the ATP binding site, which may account for its previous characterization as a competitive inhibitor. However, based on our cellular studies the likely mode of action within our experimental design is through interfacial binding near E470, and mutations at E470 would likely alter binding of CB-5083. We also performed docking studies on NMS-873 because our resistant cell lines O-CB-R1 and O-CB-R2 displayed cross-resistance (Figure 2c). NMS-873 has been previously shown to be an allosteric inhibitor of VCP and binds at the interface between the D1 and D2 domains.^30^ Molecular docking of this compound into hexameric VCP with the same search parameters outlined for CB-5083 yielded binding modes at the interface (−9.9 kcal/mol) with direct contacts to E470 (Figures 5d and e). This is consistent with mutations at E470 promoting resistance due to disruption of NMS-873 binding.

## Discussion

We observed acquired mutations in *VCP* in ovarian cancer cells upon the acquisition of *in vitro* resistance to CB-5083. In particular, we consistently observed mutations at E470 in the resistant clones. Our results also indicate that specific substitutions, such as E470K or E470D, enhance the *in vitro* ATPase activity. Consistent with enhanced ATPase activities of these mutants, higher concentrations of CB-5083 were required to inhibit the activities of these mutants. Although these studies highlight the specificity of VCP inhibition by CB-5083 and the cytotoxic effect produced by VCP inhibition, our studies also provide an important cautionary note in the development of novel therapeutics targeting VCP. Cancer cells can rapidly acquire activating mutations in *VCP* that could bypass the effect of CB-5083.

Codon 470 is located in the linker region between D1 and D2 domains of VCP. Utilizing Cryo-EM, previous studies solved the structure of VCP bound to another inhibitor UPCDC30245, and the results indicate that UPCDC30245 binds to the linker region between D1 and D2 domains.^2^ This interaction interferes with the two-step sequential activation of D2 and D1 by ATP binding. Docking of CB-5083 to the structure, reported by Banerjee *et al.*, suggests that CB-5083 may also associate with the D1–D2 linker region as well as the active site. Therefore, we speculate that the mechanism of inhibition of VCP by CB-5083 may be similar to UPCDC30245, in which binding of a ligand to the interface of the two domains prevents the ratcheting motion, between D1 and D2 domains, that is required for the function of a full hexamer.^2^ Future structural studies investigating the interaction between VCP and CB-5083 are needed to confirm this assertion.

Docking studies suggest that, in addition to binding at the active site of VCP, CB-5083 may bind to the linker region between D1 and D2 and interact with residue 470. E470K substitution could, therefore, disrupt the hydrophobic interaction between CB-5083 and E470 and interfere with that binding mode. Although the full effect of the mutation cannot be completely understood by docking alone, it is possible that amino acid substitution partially occludes the CB-5083 binding site, making CB-5083 binding less thermodynamically favorable. Therefore, we speculate that E470 substitutions reduce the affinity to CB-5083 and promote resistance to CB-5083.

**Figure 6:**
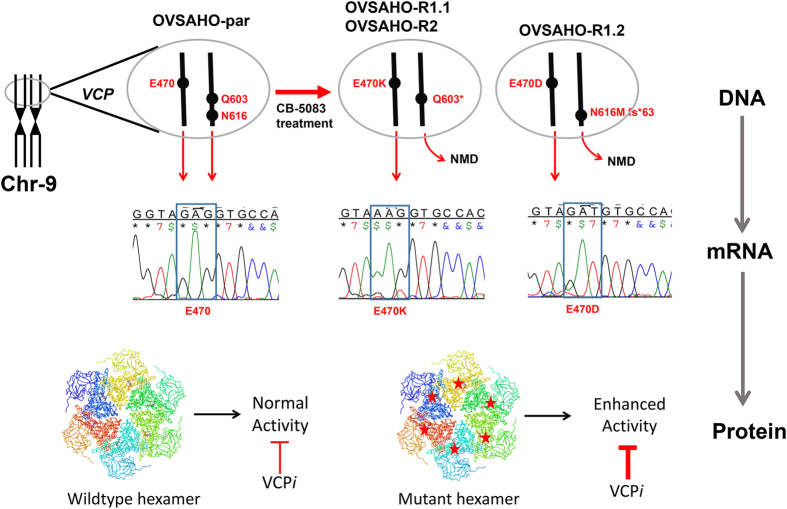
Graphical abstract elucidating the *in vitro* mechanism of resistance to CB-5083. Incremental and prolonged exposure to CB-5083 produced cells that acquired resistance to CB-5058. These cells harbor activating missense mutations at codon 470 (E470K or E470D) of VCP gene in one allele and inactivating nonsense (Q603*) or frameshift stop (N616Mfs*63) mutations in another allele. Although heterozygous mutations in DNA are detected at these sites, only homozygous mutations at codon 470 are detected in cDNA, suggesting that inactivating mutations are subjected to nonsense-mediated decay (NMD). The E470 mutants (E470K/E470D) display enhanced ATPase activity and require higher concentrations of VCP inhibitors to achieve the inhibitory effect.

In addition to the potential direct effect of E470 mutations on the ligand and target interactions, two other indirect mechanisms of inhibition could also be speculated. First, mutations at E470 induce confirmation changes that lower binding affinity to VCP inhibitors. Second, mutations at E470 increase the enzymatic activity of VCP, and therefore a higher amount of inhibitors is needed to inhibit VCP mutants. Results from enzymatic *in vitro* ATPase assays indicate VCP mutants are more active than the wild-type VCP. Therefore, activating mutations may contribute to resistance to VCP inhibitors (Figure 6). These results are similar to the resistance mechanism associated with kinase inhibitors where target proteins or other components of the targeted pathway became mutated to create a bypass mechanism that produces resistance to kinase inhibitors.^31^

It is interesting to note that although E470K and E470D VCP mutants are resistant to ML240, as indicated by *in vitro* ATPase assays, cells with VCP mutants are still sensitive to ML240. One possible interpretation of the results is that cytotoxicity effect produced by ML240 is not solely dependent on VCP inhibition. These results suggest that additional targets of ML240 contribute to cytotoxicity. It will be important to identify these additional targets so that ML240 can be further developed as cancer therapeutics to overcome resistance to CB-5083.

We also observed that CB-5083-resistant cells harbor two different mutations on separate alleles: (1) an activating mutations at E470 and (2) an inactivating nonsense mutation at Q603* or a frameshift deletion at N616. Complementary DNA sequence analysis of expressed VCP transcripts identified the E470 mutant transcripts as the primary transcripts. Transcripts containing Q603* and N616Mfs*63 are undetectable in the cDNA sequence analysis although these mutations are observed in the DNA sequence analysis. These results support our conclusion that nonsense mutations (Q603* and N616Mfs*63) are located in the other *VCP* allele and that transcripts produced from this mutant VCP are subjected to nonsense-mediated decay. Additionally, cells with mutant VCP show reduced expression of VCP, further providing a corroborating evidence that one allele is not expressed. Collectively, these results suggest a co-selection of activating and inactivating mutations in *VCP* under *in vitro* CB-5083 selection pressure.

Finally, we observed inactivating mutations in *VCP* in tumor samples reported by the Cancer Genome Atlas (TCGA) sequencing studies. In particular, truncating mutations at codon 616 are the most frequent hotspot mutations in *VCP* in TCGA database. Given that our *in vitro* selection with CB-5083 resulted in co-selection of activating and inactivating mutations in separate *VCP* alleles, the pre-existence of truncating mutations at codon 616 may portend acquired resistance to CB-5083. Therefore, ongoing clinical trials investigating the therapeutic effect of CB-5083 in cancer should investigate the theranostic potential of these mutations in cancer patients enrolled in the clinical trials.

## Materials and methods

### Cell lines and cell culture

Parental and resistant cells from OVSAHO cell were cultured in RPMI 1640 (Caisson Labs, Smithfield, UT, USA, RPL03) with 10% fetal bovine serum (Sigma-Aldrich, St. Louis, MO, USA, F0926) with 1% penicillin–streptomycin solution (Caisson Labs, PSL01) using standard sterile cell culture protocol. Cells were incubated in a humidified incubator with 5% CO_2_ at 37 °C. All cell lines were periodically checked for mycoplasma contamination. The identity of the cell lines was verified by STR genotyping (Supplementary Document).

### Reagents and chemicals

CB-5083 (Selleckchem, Houston, TX, USA, S8101), ML240 (Sigma-Aldrich, SML1071), DBeQ (Sigma-Aldrich, SML0031), NMS-873 (Selleckchem, S7285) and UPCDC30245 (Sigma-Aldrich, SML1674) were dissolved in DMSO to make a 50 mM stock solution. Tunicamycin (Sigma-Aldrich, T7765) was dissolved in DMSO to make 10 mM stock solution. The stock solution was aliquoted and stored at −80 °C, which was further diluted before making the final concentrations of the compound in culture media.

### Sulforhodamaine B assay

Cell viability following drug treatment was accessed using Sulforhodamine B (SRB) assay and performed as previously described^32^ with certain modifications. 10 000 cells/well were seeded in a 96-well plate and were allowed to incubate overnight in the humidified incubator. The following day, media was aspirated from each well and cells were incubated with fresh media containing different concentrations of the specified compound and further incubated at 37 °C incubator for 72 h.

After drug treatment, the medium was aspirated and cells were fixed with 10% trichloroacetic acid solution (Sigma-Aldrich, T6399) at 4 °C overnight. Plates were further processed as previously described.^27^ Dose–response curves were generated using GraphPad Prism 6 (La Jolla, CA, USA). Before obtaining GI_50_ values, curves were constrained at 100% on top and greater than 0% on bottom.

### Colony formation assay

Colony formation assay was performed as previously described^27^ with certain modifications. Briefly, 500 cells were plated in a six-well plate and incubated overnight at 37 °C in an incubator. Next day, cells were treated with different concentrations of CB-5083 and incubated for 48 h. The drug-containing media was aspirated, washed with PBS and replaced with regular media. Cells were then placed back in the incubator and allowed to form colonies for 6–8 days additional days replacing media every 2 days.

After the establishment of colonies, media was aspirated and cells were washed with PBS and stained with SRB dye for 30 min. Excess dye was removed and the wells were washed with 1% acetic acid. Plates were allowed to air-dry at room temperature and each plate was photographed to display the colonies using the Bio-Rad Imager System (Hercules, CA, USA).

### Western blot

0.5×10^6^ cells/well were plated in a six-well plate and incubated overnight using standard cell culture practice. Next day, media was aspirated and cells were washed twice with PBS. Cells were then incubated with media containing the compound of interest or the vehicle for the mentioned time. At the end of treatment, cells were collected using a cell scraper. To collect the cell pellet, the mixtures were centrifuged at 1000×*g* for 5 min at room temperature and subsequently washed with PBS.

Whole-cell lysates were prepared and western blot analyses were performed according to the previously established protocol.^27^ Original western blot images are provided in Supplementary Figure S6. A complete list of primary antibodies and dilutions is provided in Supplementary Figure S3B.

### Genomic DNA extraction and Sanger sequencing

1×10^6^ cells were collected from parental and resistant cell lines. Genomic DNA was extracted from each sample using Qiagen DNA purification kit (Qiagen, Hilden, Germany, 69506). Following the genomic DNA purification, segments of the VCP gene (exons 11–16) were amplified by PCR with specific primers. A complete list of all primers can be found in Supplementary Figure S3A. Following PCR amplification, amplicon length was evaluated using DNA gel electrophoresis. All samples were PCR purified (Qiagen, 28104) and sequenced by Sanger sequencing according to GeneWiz (South Plainfield, NJ, USA) PCR purified protocol. All Sanger sequencing results were then evaluated using Sequencher (ver 5.0, Gene Codes).

### RNA extraction, quantitative RT-PCR and cDNA sequencing

1×10^6^ cells pellets from each experimental conditions were harvested by centrifugation after washing with PBS. RNA extraction was performed using trizol reagent (Invitrogen, Carlsbad, CA, USA, 15596-028) according to the manufacturer’s protocol. One microgram of total RNA was reverse transcribed using a random primer (5 *μ*g) and MMLV reverse transcriptase (Invitrogen) according to the manufacturer’s protocol.

For quantitative RT-PCR, 0.5% of the cDNA reaction mixture was mixed with Qiagen qPCR SYBR Green Fluor Mastermix (Qiagen, 330513) based on the manufacturer’s protocol. The list of primers used for qRT-PCR can be found in Supplementary Figure S3D.

cDNA sequence spanning exon 7 -3′ UTR of VCP was amplified using the specific primer pairs (Supplementary Figure S3C) and the resulting amplicon was PCR purified with PCR Purification kit (Qiagen, 28104). Purified amplicons were sequenced by Sanger sequencing according to the GeneWiz PCR purified protocol. The results were then evaluated using Sequencher (ver 5.0, Gene Codes).

### Determining IC_50_ values of p97 inhibitors in ATPase assays

The detailed method was described previously.^28^ Inhibition of human p97 (25 nM monomer) was carried out in assay buffer (50 mM Tris pH 7.4, 20 mM MgCl_2_, 1 mM EDTA, 0.5 mM TCEP) containing 0.01% Triton X-100 and 200 *μ*M ATP. Eight-dose titration was used to determine IC_50_ of each compound in blocking ATPase activity, which was determined through the addition of Biomol Green Reagent (Enzo Life Sciences, Farmingdale, NY, USA).

### Molecular docking

All molecular docking experiments were carried out with AutoDock Vina.^29^ The structure of VCP was acquired from the protein data bank (PDB code 5FTK), and modified by removing the bound ADP and solvent molecules, and converted to a hexameric form for CB-5083 and NMS-873.^2^ Auto-DockTools 4.2 was utilized to add polar hydrogens and Gasteiger charges and to position a 30×30×30 Å^3^ search box that included both E470 and the active site.^33^ Structure coordinates of compounds of interest were built using Phenix and then converted to a.pdbqt format with Auto-DockTools 4.2, allowing full ligand flexibility.^34^ Default Autodock Vina settings were utilized for the analysis of binding modes, except that exhaustiveness was raised to 100. Measurements and figures were made in PyMol.^35^

### Analysis of somatic mutations in tumor samples from the Cancer Genome Atlas (TCGA) database

The database was assessed through the cbioportal website.^36^ All TCGA tumor sequencing studies (53 published and unpublished studies) were selected. The search for VCP genes indicates a total of 139 mutations (111 misense and 28 truncating mutations) in 79 samples. Thirteen samples have two mutations per sample, and the majority of these mutations have differences in variant allele frequencies, suggestive of independent or separate mutations.

## Additional information

**Publisher’s note:** Springer Nature remains neutral with regard to jurisdictional claims in published maps and institutional affiliations.
