# Supplementary material for: Specific mutations in the D1–D2 linker region of VCP/p97 enhance ATPase activity and confer resistance to VCP inhibitors
Source: Cell Death Discov. 2017 Nov 6;3:17065–. doi: 10.1038/cddiscovery.2017.65 (PMC5672561; doi:10.1038/cddiscovery.2017.65)
Supplement: Supplementary Figures [file cddiscovery201765-s1.pdf]

Supplementary-1

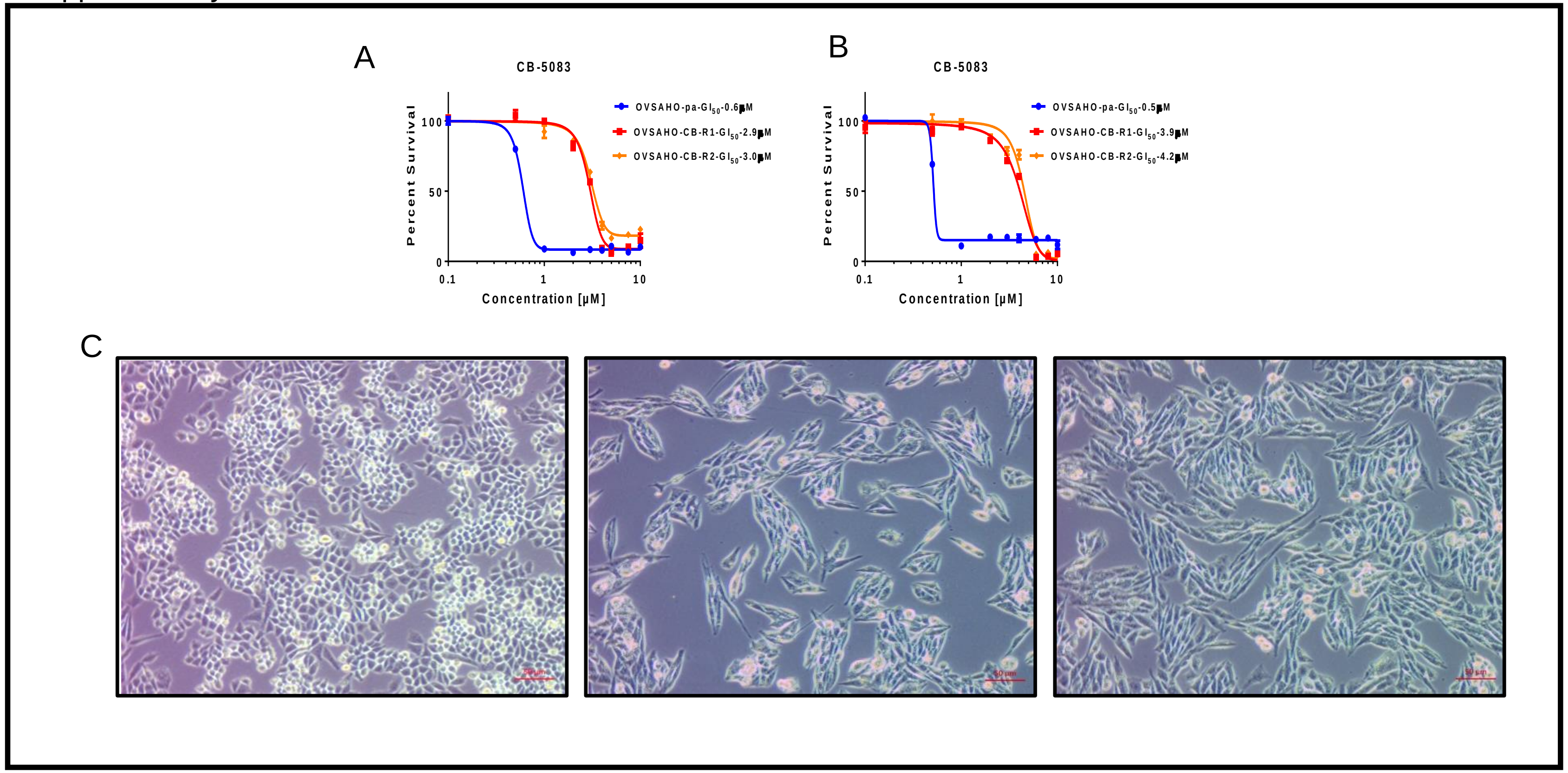

Supplementary 1: (A&B) OVSAHO parental, OVSAHO-CB-R1 and OVSAHO-CB-R2 were treated with different concentrations of CB-5083 up to 10µM. Cell viability was accessed using SRB assay at 72 hours time-point. Survival plots were generated with GraphPad prism using four parameters nonlinear regression and the curves were constrained on top (100%) and bottom (>0%). Every point in the dose response curve represents Mean± SEM taken from at least duplicate samples for all cell lines. C) Uncropped images of OVSAHO parental, O-CB-R1 and O-CB-R2 (shown in Figure 1F) taken on Zeiss light microscope at 100X magnification.

Supplementary-2

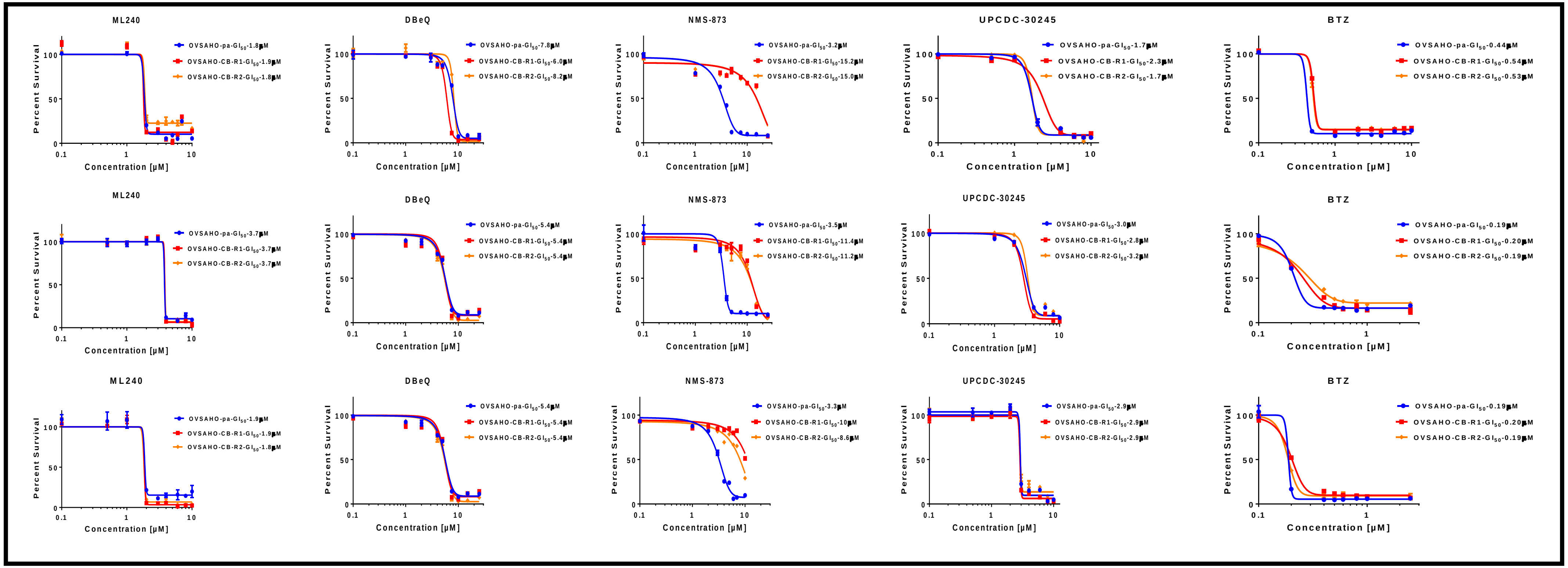

Supplementary 2: OVSAHO parental, OVSAHO-CB-R1 (O-CB-R1) and OVSAHO-CB-R2 (O-CB-R2) were treated with different concentrations of compounds up to the indicated concentrations. Cell viability was accessed using SRB assay at 72 hours time-point. Survival plots were generated with GraphPad prism using four parameters nonlinear regression and the curves were constrained on top (100%) and bottom (>0%). Every point in the dose response curve represents Mean± SEM taken from at least duplicate samples for all cell lines.

Supplementary-3

A

List of sequencing primers Exons 11-16 (VCP-gene)			
**Primers**	**Forward primer 5'-3'**	**Reverse primer 5'-3'**	**Amplicon**
VCP-Ex-11_12	TGGGTCTTTGAGGCAGCATA	TGACTCACCCTGGACCAAGT	449
VCP-Ex-13	TAATGGAGGGGATGCTTCTG	GCCCTCAGGCAAATCAATAC	339
VCP-Ex-14	CATGCTGGTTTCGGATTTCT	GCCTGAGGACTCATGCAAGT	498
VCP-Ex-15	GGGTTGGTCTAAAGGGAAGG	TCTCCATGATTGGCACATCT	375
VCP-Ex-16	TTTCCAGAGTGCATTGACAAGT	TTTGGTGTAGGTCCCCAAAG	399

B

List of Antibodies			
**Antibody**	**Company**	**Catalog Number**	**Dilution Used**
ATF4	Cell Signaling Technology	11815S	1/1000
VCP	Santa-Cruz Biotechnology	20799	1/1000
β-Actin	Sigma-Aldrich	A1978	1/5000

C

For cDNA sequencing			
**Primers**	**Forward primer 5-3**	**Reverse primer 5-3**	**Amplicon**
VCP_cDNA	CAATTGGTGTGAAGCCTCCT	AGAAACCCCCTGTCCAGAGT	1886

D

For q-RT-PCR			
**Primers**	**Forward primer 5-3**	**Reverse primer 5-3**	**Amplicon**
VCP_cDNA	ATGCCATCGCTCCCAAAAGA	TGTCAAAGCGACCAAATCGC	174
18S	GCCCGAAGCGTTTACTTTGA	TCCATTATTCCTAGCTGCGGTATC	81

Supplementary-3: A list of PCR primers and antibodies

Supplementary-4

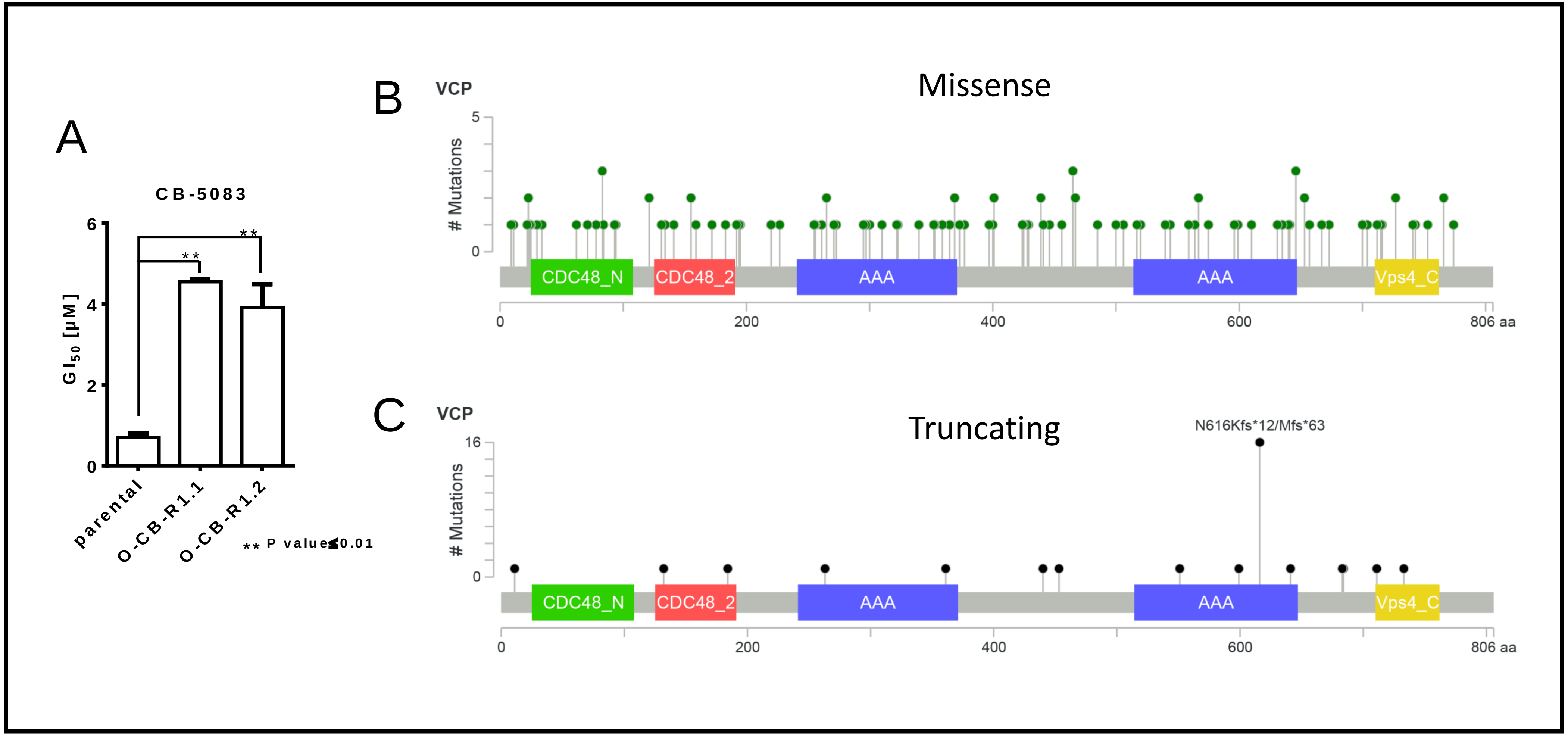

Supplementary 4: A) The bar graph represents Mean GI_{50} ± SEM in OVSAHO-parental and two pure clones from O-CB-R1 (O-CB-R1.1 and O-CB-R1.2) taken from three independent experiments. P-values were calculate using Student's t-test. Known missense mutations (B) and truncation mutations (C) associated with cancer.

Supplementary-5

A B

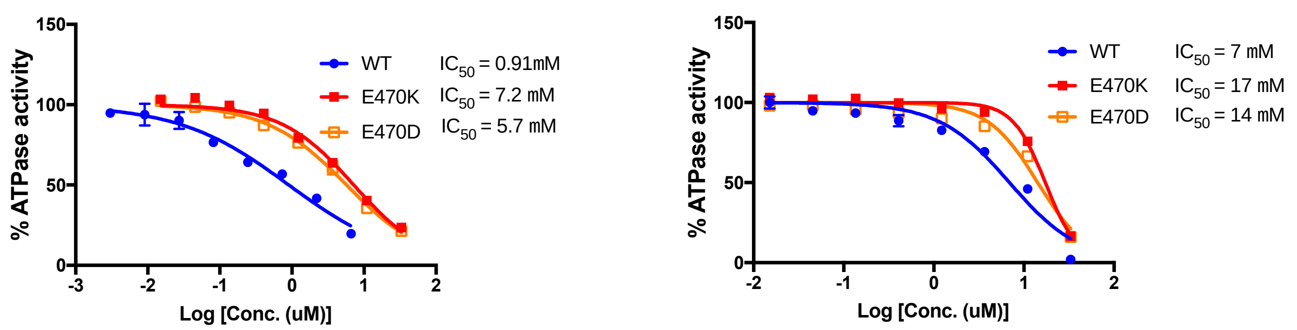

C

Plasmids used in this study

Plasmid Number	Plasmid name	Vector	Source and Reference
**TCB-197/ SLB-001**	Human p97 pET15_T	pET15b_TEV linker	Chou, 2014*
**TCB-499**	E470D human p97 pET15_T	pET15b_TEV linker	This study
**TCB-490**	E470K human p97 pET15_T	pET15b_TEV linker	This study

*Chou, T. F., Bulfer, S. L., Weihl, C. C., Li, K., Lis, L. G., Walters, M. A., Schoenen, F. J., Lin, H. J., Deshaies, R. J., and Arkin, M. R. (2014) Specific inhibition of p97/VCP ATPase and kinetic analysis demonstrate interaction between D1 and D2 ATPase domains. *J. Mol. Biol.* **426**, 2886-2899.

Supplementary 5:Titration curves of ML241 (A) and DBeQ (B) for WT, E470K and E470D VCP proteins in inhibiting ATPase activity. IC_{50} (uM) was calculated using Prism with the equation [log(agonist) vs. response -- Variable slope (four parameters)].

Supplementary-6

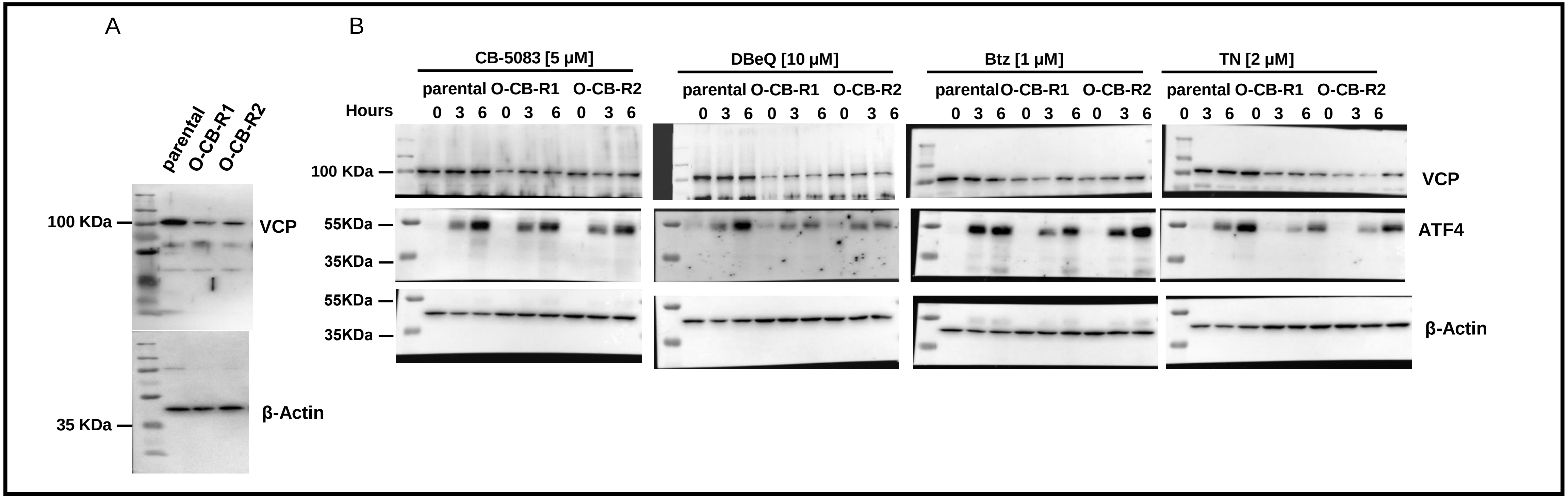

Supplementary 6: Original western blot images shown in Figure 2.
